# Supplementary material for: Unravelling the Effect of Provitamin A Enrichment on Agronomic Performance of Tropical Maize Hybrids
Source: Plants (Basel). 2021 Jul 31;10(8):1580. doi: 10.3390/plants10081580 (PMC8398423; doi:10.3390/plants10081580)
Supplement: Supplementary file 1 [file plants-10-01580-s001.zip › plants-1268362-supplementary.pdf]

Supplementary Table S1. The list of hybrids included in the regional trials evaluated across 23 environments for carotenoids and 99 environments for agronomic traits.

Hybrids	Pedigree	Type of hybrids
H01	(KU1409/KU1414-SR/A619)-S2-2/9450xKI21-7-2-2-1-1-BB//IITATZII528	Three-way cross
H02	(KU1409/DE3/KU1409) S2-27-B*7/(9450xKI28)-1-2-1-2-B*4//IITATZII528	Three-way cross
H03	IITATZII528/(KU1409/DE3/KU1409) S2-28-B*4//(ACR97TZL-CCOMP1-Y-S3-12-2-BB/CI7/ACR97TZL-CCOMP1-Y-S3-12-2-BB)-13-B	Three-way cross
H04	IITATZII528/(9450xKI21-1-4-1-1-2-B/DE3/9450xKI21-1-4-1-1-2-B)-9-B*4//(KU1409/SC55/KU1409)-S2-38-1-B*5	Three-way cross
H05	IITATZII528/9450xKI21-1-4-1-1-1-B/DE3/9450xKI21-1-4-1-1-1-B-23-B*5//(KU1409/KU1414-SR/A619)-S2-6-B*5	Three-way cross
H06	IITATZII528/(9450xKI21-1-5-2-1-2-B/DE3/9450xKI21-1-5-2-1-2-B)-22-B*4//(KU1409/KU1414-SR/A619)-S2-5-B*5	Three-way cross
H07	IITATZI1729/IITATZI1748//IITATZII528	Three-way cross
H08	(9450xKI21-3-2-2-1-2-B/CI7/9450xKI21-3-2-2-1-2-B)-11-B*4/(KU1409/DE3/KU1409) S2-28-B*6//IITATZII528	Three-way cross
H09	(KU1409/DE3/KU1409) S2-11-B*7/(9450xKI28)-1-2-1-2-B*4//IITATZII528	Three-way cross
H10	IITATZII528/(KU1409/KU1414-SR/A619)-S2-3-B//9450xKI 21-1-4-1-1-2-B-B-B-B-B	Three-way cross
H11	KU1409/NC358/KU1409-17-B*8/(POP66SR/ACR91SUWAN1-SRC1/ACR91SUWAN1-SRC1-6x(MP420x4001xMP420)-3-1-3-1-B) S2-10-B*7//IITATZII528	Three-way cross
H12	(KU1409/KU1414-SR/KVI3)-S2-5-1-BBB/9450xKI21-3-1-1-2-1-B*5//IITATZII528	Three-way cross
H13	KU1409/SC55/KU1409-4-B*6/(9450xKI28)-1-2-1-2-B*4//IITATZII528	Three-way cross
H14	(SYN-Y-STR-34-1-1-1-1-2-1-B*5/NC354/SYN-Y-STR-34-1-1-1-1-2-1-B*5)-S2-7-5-BB-B-B-B-B/(KU1409/KU1414-SR/NC350)-S2-1-2-BB//(9450xCM116x9450)-5-2-3-1-B*4	Three-way cross
H15	((9071x4058)-8-2-1-1-B*5/TZMI214xA619LPAxTZMI214-9-3-B*5)-38-1-BBB/(KU1409/DE3/KU1409)S2-18-2-BB/TZMI214xA619LPAxTZMI214-26-2-B*5-36-2-B// IITATZII528	Three-way cross
H16	((9071x4058)-8-2-1-1-B*5/TZMI214xA619LPAxTZMI214-9-3-B*5)-43-1-BBB/((KU1409/DE3/KU1409)S2-7-BBB/TZMI214xA619LPAxTZMI214-9-3-B*5)-27-2-B// IITATZII528	Three-way cross
H17	IITATZII528/(KU1409/DE3/KU1409) S2-28-1-B*7//(ACR97TZL-CCOMP1-Y-S3-12-2-BB/CI7/ACR97TZL-CCOMP1-Y-S3-12-2-BB)-15-B*4	Three-way cross
H18	(KU1409/DE3/KU1409) S2-28-B*4/(ACR97TZL-CCOMP1-Y-S3-12-2-BB/CI7/ACR97TZL-CCOMP1-Y-S3-12-2-BB)-13-B/(POP66SR/ACR91SUWAN1-SRC1//IITATZII528	Three-way cross

H19	(KU1409/DE3/KU1409) S2-15-B*7/9450xKI21-1-5-3-2-2-B*8//IITATZI1527	Three-way cross
H20	(KU1409/DE3/KU1409) S2-36-B*6/(4001/9848/4001)-10-1-2-B*5//IITATZI1527	Three-way cross
H21	(KU1414-SR/CI7/KU1414-SR)-63-B*4/(9450xKI28)-1-2-1-1-B/DE3/(9450xKI28)-1-2-1-1-B-29-B*5//IITATZI1527	Three-way cross
H22	(KU1414-SR/CI7/KU1414-SR)-63-B*4-B/(9450xKI21-1-4-1-1-2-B/DE3/9450xKI21-1-4-1-1-2-B)-29-B*5/IITATZI1527	Three-way cross
H23	(KU1409/DE3/KU1409) S2-27-BB/(POP66SR/ACR91SUWAN1-SRC1/ACR91SUWAN1-SRC1-8XPOP61-SR-11-2-3-3-1-B) S2-4-B*5//IITATZII528	Three-way cross
H24	KU1409/SC55/KU1409-4-B-B-B-B(9450xCM116x9450)-3-3-1-2-1-B*6//IITATZI1527	Three-way cross
H25	IITATZI1716/IITATZII528	Single-cross
H26	(4205/CI7/4205)-6-B*6/(9450/KI21-1-4-1-1-2-B/DE3/9450xKI21-1-4-1-1-2-B)-29-BBB//IITATZI1715	Three-way cross
H27	(KU1409/DE3/KU1409) S2-9-B*6/(POP66SR/ACR91SUWAN1-SRC1/ACR91SUWAN1-SRC1-6X(MP420x4001xMP420)-3-1-3-1-B)S2-10-B*7//IITATZI1527	Three-way cross
H28	A0905-28/IITATZI1629	Three-way cross
H29	A0905-28/(KU1409/DE3/KU1409) S2-18-2-BBB/(KU1409/SC55/KU1409)-S2-19-1-B-B-B-12-B-1-B	Three-way cross
H30	A0905-28/(KU1409/DE3/KU1409) S2-18-2-BBB/SC55/KU1414-SR/KU1414-SR-6-B*5-1-B-2-B	Three-way cross
H31	A0905-28/(KU1409/KU1414-SR/KVI3)-S2-8-1-B*5/(KU1409/DE3/KU1409) S2-21-B*4-11-B-1-B	Three-way cross
H32	A0905-28/KS 23 (S) C5-1-3-2-B	Three-way cross
H33	(KU1409/KU1414-SR/KVI3)-S2-5-1-BBB/9450xKI21-3-1-1-2-1-B*5//IITATZII528	Three-way cross
H34	(KU1409/DE3/KU1409) S2-15-B*5/9450xKI 21-1-5-3-2-2-B-B-B-B-B-B-B//IITATZI1527	Three-way cross
H35	((KU1409/DE3/KU1409)S2-7-BBB/(TZMI214xA619LPAxTZMI214-9-3-B*5)-27-1-BBB/((9071x4058)-8-2-1-1-B*5/TZMI214xA619LPAxTZMI214-9-3-B*5)-46-1-B// IITATZII528	Three-way cross
H36	IITATZI1727/IITATZI1745/IITATZII528	Three-way cross
H37	(9450/KI 21-1-4-1-1-2-B/DE3/9450/KI 21-1-4-1-1-2-B)-30-B-B-B-B/(KU1409/DE3/KU1409) S2-7-B*5//IITATZII528	Three-way cross
H38	IITATZI1727/IITATZI1566//IITATZII528	Three-way cross
H39	IITATZI1718/IITATZI1629	Single-cross
H40	IITATZI1716/IITATZI1629	Single-cross
H41	(9450xKI21-1-5-2-1-2-B/DE3/9450xKI21-1-5-2-1-2-B)-22-B*4/(KU1409/KU1414-SR/A619)-S2-5-B*5//IITATZII528	Three-way cross
H42	A0905-28/(KU1409/SC55/KU1409-4-BBB/(KU1409/DE3/KU1409)) S2-18-2-BBB-25-B-1-B	Three-way cross

H43	(KU1409/SC55/KU1409-4-BBB/(KU1409/DE3/KU1409)) S2-18-2-BBB-9-B-1-B/ACR97TZL-CCOMP1-Y-S3-33-5-B*9//(9450xKI21-1-5-2-1-2-B/DE3/9450xKI21-1-5-2-1-2-B)-9-B*5	Three-way cross
H44	(KU1409/SC55/KU1409-4-BBB/(KU1409/DE3/KU1409)) S2-18-2-BBB-25-B-1-B/ACR97TZL-CCOMP1-Y-S3-33-5-B*9//(9450xKI21-1-5-2-1-2-B/DE3/9450xKI21-1-5-2-1-2-B)-9-B*5	Three-way cross
H45	((KU1409/DE3/KU1409) S2-15-B*4/(KU1409/KU1414-SR/KVI3))-S2-8-2-B*5-3-B-1-B/IITATZI1262//IITATZI1557	Three-way cross
H46	(KU1409/SC55/KU1409-4-BBB/(KU1409/DE3/KU1409)) S2-18-2-BBB-9-B-1-B/ IITATZII528//((9450xKI21-1-5-2-1-2-B/DE3)/9450xKI21-1-5-2-1-2-B))-9-B*5	Three-way cross
H47	(KU1409/DE3/KU1409) S2-2-BB/(9450xCM116x9450)-3-3-1-2-1-B*5//IITATZI1522	Three-way cross
H48	(KU1409/KU1414-SR/A619)-S2-2/9450xKI21-7-2-2-1-1-BB//IITATZII528	Three-way cross
H49	((9071x4058)-8-2-1-1-B*5/TZMI214xA619LPAxTZMI214-9-3-B*5)-38-1-B/IITATZII528//IITATZI1716	Three-way cross
H50	(9450xKI21-1-5-2-1-2-B/DE3/9450xKI21-1-5-2-1-2-B)-22-B*5/(KU1409/KU1414-SR/A619)-S2-5-B*5//IITATZII528	Three-way cross
H51	(9450xKI21-1-5-2-1-2-B/DE3/9450xKI21-1-5-2-1-2-B)-22-B*5/(KU1409/KU1414-SR/A619)-S2-5-B*5//IITATZII528	Three-way cross
H52	(POP66SR/ACR91SUWAN1-SRC1/ACR91SUWAN1-SRC1-6X(MP420x4001xMP420)-3-1-3-1-B) S2-1-B*7//IITATZI1800	Three-way cross
H53	IITATZI1653/IITATZI1262//IITATZI1629	Three-way cross
H54	((KU1409/KU1414-SR/A619)-S2-2/(9450/KI21-7-2-2-1-1-BB))-28-B*4-B-1/IITATZI1715//IITATZI1546	Three-way cross
H55	A0905-28//(KU1409/DE3/KU1409) S2-18-2-BBB/(KU1409/SC55/KU1409)-S2-19-1-BBB-12-B-1-B	Three-way cross
H56	A0905-28//((KU1409/DE3/KU1409)S2-18-2-BBB/(KU1409/SC55/KU1409)) S2-19-1-BBB-17-B-1-B	Three-way cross
H57	A0905-28//((KU1409/KU1414-SR/KVI3)-S2-8-1-B*5/(KU1409/DE3/KU1409)) S2-21-B*4-11-B-1-B	Three-way cross
H58	A0905-28//(KU1409/KU1414-SR/M162W)-S2-4-BBB/(KU1409/DE3/KU1409) S2-4-B*4-3-B-1-B	Three-way cross
H59	A0905-28//IITATZI1653	Three-way cross
H60	A0905-28//IITATZI1739	Three-way cross
H61	IITATZI1716/IITATZI1322 (Commercial provitamin A biofortified hybrid)	Single-cross
H62	IITATZI1717/IITATZI1629 (Commercial provitamin A biofortified hybrid)	Single-cross
H63	Oba Super-II (Orange commercial benchmark hybrid)	Single-cross
H64	Farmers preferred variety	

Supplementary Table S2. Best linear unbiased estimates (BLUPs) for individual carotenoids and provitamin A recorded in 23 environments.

Hybrid	LUT	ZEAX	BCRYPTO	ACAR	BCAR	PVA	TCAR	CAN1	CAN2	Groups
	(µg/g)							(Scores)		
H02	9.5	15.8	5.4	1.4	6.0	9.5	39.8	1.42	-0.30	1
H14	8.7	15.7	5.3	1.5	5.7	8.7	40.0	1.96	0.35	1
H43	8.2	14.2	4.0	1.1	5.0	8.2	34.6	-0.09	0.18	1
H44	7.6	14.3	4.1	1.1	4.1	7.6	35.0	-0.71	-1.75	1
H45	7.9	13.9	4.1	1.1	4.6	7.9	35.0	0.11	-0.84	1
H46	8.6	16.3	4.6	1.3	5.1	8.6	39.1	-1.48	0.03	1
H63	8.8	17.3	5.2	1.3	4.8	8.8	37.1	0.18	-2.72	1
H64	7.9	16.2	4.6	1.2	4.7	7.9	35.0	0.71	3.03	1
H01	11.4	16.5	5.9	1.7	7.8	11.4	42.0	0.63	0.80	2
H03	10.0	17.9	5.9	1.6	6.3	10.0	40.8	-1.43	1.01	2
H04	9.8	16.6	6.0	1.6	6.0	9.8	40.1	-0.70	-0.26	2
H05	10.4	16.4	6.3	1.6	6.4	10.4	40.9	-1.11	0.12	2
H07	11.2	17.1	5.4	1.4	7.8	11.2	40.4	1.11	0.62	2
H12	10.5	18.2	6.2	1.5	6.6	10.5	42.7	1.36	1.17	2
H13	12.0	16.8	6.5	1.5	7.5	12.0	41.2	0.82	0.16	2
H17	10.6	16.2	6.2	1.5	6.7	10.6	39.4	-1.44	1.55	2
H18	11.1	17.1	6.4	1.7	6.4	11.1	42.7	2.32	-0.07	2
H21	11.9	15.8	6.6	1.6	7.9	11.9	41.3	0.28	0.75	2
H22	11.8	17.4	6.2	1.6	8.0	11.8	42.1	0.83	0.72	2
H23	11.0	16.6	7.0	1.8	6.7	11.0	42.1	2.13	1.20	2
H24	12.1	17.6	6.3	1.7	8.2	12.1	43.3	1.20	-1.25	2
H25	12.0	15.0	6.7	1.8	7.9	12.0	39.3	1.39	-1.07	2
H26	11.4	15.2	4.2	1.2	8.8	11.4	38.4	0.87	-0.65	2
H28	10.6	17.5	6.3	1.6	6.6	10.6	42.6	1.06	-2.82	2
H29	10.7	17.3	6.4	1.6	6.5	10.7	40.4	0.20	-0.83	2
H31	10.4	16.3	6.4	1.6	6.3	10.4	40.2	0.20	0.62	2
H32	9.9	17.8	5.3	1.4	6.6	9.9	40.6	-0.49	-1.13	2
H36	12.5	18.4	6.5	1.6	8.5	12.5	44.1	2.50	1.84	2
H37	10.6	17.2	5.9	1.6	6.5	10.6	39.9	-4.68	-0.33	2
H39	10.1	17.0	6.1	1.5	6.8	10.1	41.6	0.89	1.17	2
H40	11.2	15.9	5.3	1.5	7.1	11.2	37.9	2.26	-0.83	2
H41	10.8	16.7	6.2	1.7	6.8	10.8	40.6	-0.67	-0.81	2
H42	9.9	16.1	5.7	1.5	5.9	9.9	39.2	0.63	1.06	2
H47	10.1	15.0	5.1	1.3	6.8	10.1	36.0	-4.80	0.86	2
H49	11.0	16.7	7.0	1.6	6.3	11.0	39.2	-5.01	1.53	2
H51	9.8	18.5	4.5	1.4	7.1	9.8	41.4	-2.09	-1.78	2
H52	11.0	13.7	6.5	1.6	6.8	11.0	37.2	0.69	0.71	2
H54	10.3	17.5	5.5	1.5	6.6	10.3	40.7	1.87	-0.46	2
H55	11.6	17.4	6.5	1.6	7.1	11.6	41.1	-0.18	0.91	2
H56	10.5	17.8	6.2	1.6	6.6	10.5	39.9	-1.03	-2.21	2
H57	11.1	16.3	6.1	1.6	6.6	11.1	40.3	2.01	0.85	2

H58	10.8	14.2	5.5	1.5	7.7	10.8	37.4	-0.56	0.06	2
H59	10.7	14.1	5.9	1.5	8.2	10.7	37.6	0.36	-1.36	2
H61	11.6	17.7	7.1	1.9	7.2	11.6	42.5	-0.41	-0.43	2
H06	10.8	18.9	6.0	1.6	7.1	10.8	43.9	-0.40	-0.03	3
H08	11.0	16.4	6.3	1.7	7.1	11.0	42.9	0.29	-0.98	3
H09	11.2	17.4	6.6	1.7	6.9	11.2	44.4	0.92	0.69	3
H10	11.2	16.3	5.1	1.5	8.0	11.2	41.1	-3.68	-0.27	3
H11	11.5	19.3	6.5	1.8	7.3	11.5	45.1	1.51	-1.55	3
H15	14.0	19.0	8.4	1.6	7.8	14.0	47.2	0.03	0.54	3
H16	10.3	17.4	6.0	1.6	6.9	10.3	46.9	0.52	-1.27	3
H19	11.1	16.0	5.6	1.5	7.6	11.1	42.0	1.52	4.07	3
H20	11.1	19.8	6.8	1.8	6.9	11.1	46.4	-0.72	-1.25	3
H27	11.1	19.3	6.4	1.7	7.7	11.1	44.8	2.12	-0.73	3
H30	11.5	20.5	6.6	1.7	7.3	11.5	48.7	2.18	-0.07	3
H33	11.5	18.9	6.2	1.7	7.7	11.5	46.5	2.72	2.62	3
H34	11.3	17.1	6.6	1.7	7.5	11.3	44.2	-0.58	-0.67	3
H35	10.1	16.6	5.3	1.6	7.4	10.1	42.3	-0.89	0.28	3
H38	10.3	19.3	6.4	1.5	6.8	10.3	44.6	1.66	0.67	3
H48	10.2	19.4	5.8	1.5	7.1	10.2	43.8	-5.38	1.87	3
H50	11.7	19.1	6.6	1.7	7.6	11.7	44.3	-2.97	2.24	3
H53	11.6	16.3	5.1	2.1	7.1	11.6	42.1	-0.98	-2.16	3
H60	11.6	16.5	5.8	1.6	6.5	11.6	44.3	-0.65	-1.50	3
H62	10.7	15.2	6.1	1.7	6.9	10.7	41.8	-0.31	-1.89	3

LUT=Lutein, ZEAX= Zeaxanthin, BCRYPTO=β-cryptoxanthin, ACAR=α-carotene, BCAR=β-carotene, PVA=Provitamin A, TCA=Total Carotenoid, CAN1=first and CAN2=second canonical discriminant functions

Supplementary Table S3. Best linear unbiased estimates (BLUPs) for Provitamin A content and grain yield as well as their latent regression lines slopes obtained from factor analysis of order three (FA(3)) model fitted to the data sets recorded in 23 environments for provitamin A and 99 environments for grain yield.

Hybrid	BLUPs (μg/g)	Factor 1	Factor 2	Factor 3	BLUPs (kg/ha)	Factor 1	Factor 2	Factor 3	Groups
H44	7.6	-2.8	1.8	-2.0	3805	0.1	0.1	1.2	1
H45	7.9	-2.4	1.2	-2.4	4318	0.6	0.7	0.7	1
H43	8.2	-2.5	1.9	-1.5	3805	-0.2	-0.2	0.5	1
H46	8.6	-1.7	0.8	-1.6	2714	1.9	1.5	-2.3	1
H14	8.7	-0.6	0.1	-0.1	3578	1.5	0.8	-2.2	1
H02	9.5	-0.5	-0.4	-1.8	4269	1.1	2.0	0.6	1
H51	9.8	-1.5	1.7	-0.8	4114	1.1	0.4	-0.9	2
H04	9.8	-0.5	-0.5	-1.8	4073	-1.1	-0.5	0.2	2
H42	9.9	-0.8	0.7	-0.1	3288	-0.8	-1.1	0.9	2
H32	9.9	0.1	-0.6	0.1	3308	-0.6	-0.7	1.2	2
H35	10.1	0.5	-0.4	0.7	2996	-0.7	0.2	0.3	3
H47	10.1	-0.6	0.1	-1.7	3452	0.3	0.9	1.6	2
H31	10.4	0.6	-0.9	0.7	3598	-0.5	-0.9	1.1	2
H27	11.1	0.9	-1.1	-0.3	3294	2.9	1.7	-2.1	3
H10	11.2	-0.1	0.1	-0.2	3042	-1.6	-2.7	1.0	3
H40	11.2	1.8	-1.9	0.6	3300	-2.5	-4.9	0.5	2

H34	11.3	0.6	-0.5	0.4	3068	-2.0	-0.6	0.7	3
H11	11.5	0.2	-0.3	-0.6	2112	3.4	2.6	-2.4	3
H33	11.5	0.4	0.2	0.6	3609	3.0	1.6	-1.9	3
H36	12.5	0.9	-0.8	-0.2	2000	2.1	2.1	-1.7	2
H03	10.0	-0.1	-0.2	-0.4	4009	-1.2	-0.8	-0.2	2
H39	10.1	2.3	-2.8	0.8	3794	2.0	1.1	-0.9	2
H48	10.2	-0.6	0.9	0.3	3627	1.8	1.1	-1.0	3
H16	10.3	-1.6	1.4	-0.9	3968	0.1	-0.2	-1.3	3
H38	10.3	-0.2	-0.1	-0.5	4256	1.6	3.0	1.3	3
H54	10.3	0.0	-0.1	0.3	4424	0.6	0.6	1.2	2
H05	10.4	0.5	-0.5	0.8	4090	-1.1	-0.8	0.0	2
H12	10.5	-0.3	0.1	-0.4	3822	-0.3	-0.8	0.7	2
H56	10.5	0.1	-0.1	0.3	4252	2.6	1.1	-1.5	2
H28	10.6	0.2	-0.3	0.3	4210	-2.0	-1.2	0.2	2
H37	10.6	-0.8	0.8	-0.3	4355	0.8	0.1	-0.8	2
H29	10.7	-0.4	0.5	-0.4	3629	-0.7	-1.2	0.5	2
H59	10.7	-0.5	1.3	1.0	4456	0.8	1.1	0.3	2
H06	10.8	-0.5	0.6	-0.4	4293	0.0	-0.2	-0.5	3
H58	10.8	0.2	0.0	0.5	4342	1.9	0.7	-1.6	2
H52	11.0	0.7	-0.9	0.0	3771	1.1	0.6	-0.7	2
H49	11.0	0.4	-0.3	0.4	4006	2.9	1.4	-2.0	2
H20	11.1	-0.6	1.0	-0.5	3897	0.0	0.0	-0.3	3
H18	11.1	-0.6	1.2	0.6	4093	3.8	1.8	-1.6	2
H19	11.1	0.3	-0.6	-0.7	4329	-0.2	-0.8	-0.1	3
H57	11.1	0.8	-1.2	-0.4	4716	0.6	0.4	-0.3	2
H07	11.2	0.3	0.5	1.8	3653	-1.1	-0.5	0.0	2
H09	11.2	-0.4	1.0	0.3	4499	2.3	4.2	1.5	3
H26	11.4	-0.1	0.3	0.2	3752	-1.2	-2.6	0.8	2
H01	11.4	0.0	0.4	0.3	4100	-1.9	-1.0	0.3	2
H30	11.5	0.2	0.6	1.5	4099	-1.8	-1.1	0.0	3
H60	11.6	1.1	-1.6	-0.8	4570	0.5	0.5	-0.5	3
H50	11.7	0.5	-0.1	0.3	4440	0.8	0.0	-1.2	3
H22	11.8	0.1	0.4	0.0	4443	-0.2	-0.9	-0.4	2
H21	11.9	0.6	0.5	2.1	4629	0.1	-0.3	0.1	2
H13	12.0	0.4	-0.5	-0.5	3967	-0.4	-0.9	0.3	2
H25	12.0	-0.4	1.4	0.3	5111	0.9	0.7	0.8	2
H15	14.0	0.4	-0.2	-1.1	3829	0.1	0.0	-0.8	3
H17	10.6	1.4	-0.3	2.5	4803	1.6	2.3	1.3	2
H41	10.8	0.7	-0.2	2.5	4096	1.3	2.6	0.9	2
H23	11.0	0.3	-0.3	-0.5	4653	1.4	2.0	1.4	2
H08	11.0	1.0	-0.9	0.7	4293	1.1	1.9	0.9	3
H55	11.6	1.0	-0.9	0.3	4973	0.7	0.4	0.1	2
H53	11.6	1.5	-1.5	0.9	4258	0.6	0.8	1.4	3
H24	12.1	1.3	-0.6	1.6	4814	0.9	0.8	0.6	2
H61 COM-PVA	11.6	0.6	0.2	1.6	4567	0.6	0.5	0.7	2

H62 COM-PVA	10.7	1.9	-2.6	0.1	3610	-0.6	-1.1	0.9	3
H63 OR-COM	8.8	-2.4	2.3	-0.4	3051	0.3	0.7	-0.7	1
H64 LV	7.9	-1.3	-0.1	-1.8	3791	2.0	0.8	-1.0	1
